# Pulsed Wave Doppler Ultrasound Is Useful to Assess Vasomotor Response in Patients with Multiple System Atrophy and Well Correlated with Tilt Table Study

**DOI:** 10.1100/2012/548529

**Published:** 2012-01-03

**Authors:** Ke-Vin Chang, Wen-Shiang Chen, Ruey-Meei Wu, Ssu-Yuan Chen, Hsiu-Yu Shen, Ching Lan, Yen-Ho Wang

**Affiliations:** ^1^Department of Physical Medicine and Rehabilitation, National Taiwan University Hospital, Taipei 10002, Taiwan; ^2^Department of Physical Medicine and Rehabilitation, National Taiwan University College of Medicine, Taipei 10051, Taiwan; ^3^Department of Neurology, National Taiwan University Hospital and National Taiwan University College of Medicine, Taipei, Taiwan

## Abstract

The study aim was to assess sympathetic vasomotor response (SVR) by using pulsed wave Doppler (PWD) ultrasound in patients with multiple system atrophy (MSA) and correlate with the tilt table study. We recruited 18 male patients and 10 healthy men as controls. The SVR of the radial artery was evaluated by PWD, using inspiratory cough as a provocative maneuver. The response to head-up tilt was studied by a tilt table with simultaneous heart rate and blood pressure recording. The hemodynamic variables were compared between groups, and were examined by correlation analysis. Regarding SVR, MSA patients exhibited a prolonged latency and less heart rate acceleration following inspiratory cough. Compared with the tilt table test, the elevation of heart rate upon SVR was positively correlated to the increase of heart rate after head-up tilt. The correlation analysis indicated that the magnitude of blood pressure drop from supine to upright was positively associated with the SVR latency but negatively correlated with the heart rate changes upon SVR. The present study demonstrated that blunted heart rate response might explain MSA's vulnerability to postural challenge. PWD may be used to predict cardiovascular response to orthostatic stress upon head-up tilt in MSA patients.

## 1. Introduction

Multiple system atrophy (MSA), a neurodegenerative disease [1], manifests with autonomic failure and movement disorders such as Parkinsonism or cerebellar ataxia which responds poorly to levodopa [2]. Dysregulation of the autonomic and cardiovascular systems, which is common in patients with MSA, may cause orthostatic hypotension (OH). During postural challenge, the homeostasis requires prompt vasoconstriction to increase peripheral resistance and cardiac output to compensate volume depletion in the upright position. MSA is considered to affect the central and preganglionic sympathetic system [3]; it differs from pure autonomic failure which causes OH owing to inappropriate peripheral vasoconstriction resulting from impaired postganglionic adrenergic pathways [4]. Since OH severely impacts the safety and quality of life among patients with MSA, exploring the mechanism of orthostatic intolerance is important and may facilitate the development of new treatment.

 Sympathetic vasomotor response (SVR) indicates arteriolar vasoconstriction following sympathetic stimuli [5], and they are widely used to evaluate *α* adrenergic pathways in diseases presented with autonomic disturbance [6, 7]. Laser Doppler flowmetry is the first to quantify SVR through measuring the percentage decrement of skin blood flow [8], but may underestimate sympathetic reserve whenever resting vascular resistance is high. Continuous wave Doppler ultrasound has advantages over laser Doppler flowmetry by reflecting baseline and poststimuli vascular impedance through indicators like pulsatility or resistive index [9, 10]. Pulsed waved Doppler ultrasound (PWD) employed a time filter to target a certain vascular segment, overcoming the drawback of poor axial resolution using continuous wave Doppler. Our previous work demonstrated that PWD was capable to detail SVR regarding its central (e.g., heart rate) and peripheral (e.g., vascular diameter) components [11, 12]. Concerning that OH is mainly derived from deficit in sympathetic systems, the present study was intended to investigate the integrity of SVR in patients with MSA by using PWD and to explore the pathophysiology of orthostatic intolerance by correlation analysis with the tilt table study.

## 2. Methods

### 2.1. Patients

Eighteen patients were recruited from the outpatient Department of Neurology, National Taiwan University from September 2010 to March 2011. All patients met the diagnostic criteria of possible MSA established in the consensus conference in 2008 [13], comprising rapidly progressive Parkinsonism, poor responsiveness to levodopa, and at least one symptom suggesting autonomic disturbance. Only males were included due to the requirement of exposing right inguinal area for hemodynamic measurement during the tilt table test. We excluded patients with pulmonary or cardiovascular diseases. Ten healthy adults without systemic diseases or medication affecting autonomic systems were chosen as controls.

### 2.2. Sympathetic Provocative Maneuver and Pulsed Wave Doppler Settings

**Figure 1:**
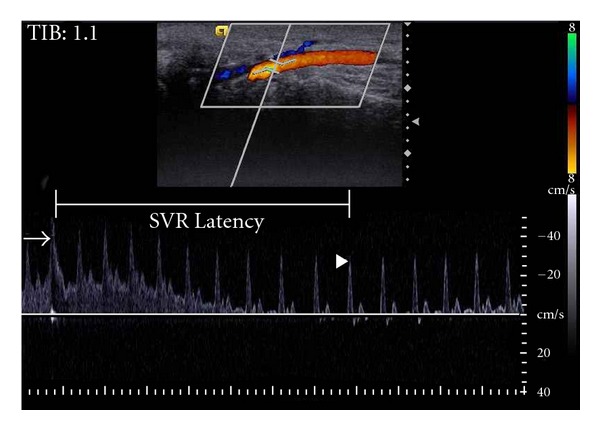
Change of Doppler wave forms upon sympathetic vasomotor response (SVR). The inspiratory cough causes an artifact (arrow) and decreases systolic blood velocity with a more prominent diastolic reversal. The wave of maximal deflection (arrowhead) was used to represent the hemodynamic status after sympathetic stimulation. The SVR latency indicated the period between the artifact by inspiratory cough to the maximal deflection of Doppler wave forms.

The participants rested quietly for 15 minutes on an armchair in an air-conditioned room set between 25°C to 28°C. The vasomotor response was measured by one experienced investigator (K.V.C.), using a pulsed wave Doppler ultrasound (Acuson S2000 system, Siemens, Munich, Germany) with a multifrequency transducer (14L5, 5–14 MHz in 2D mode and 5.5–7.5 MHz in Doppler mode). Subject's right forearm was in supination with a pillow placed under right wrist. The probe was placed on the ventral wrist to parallel the long axis of the forearm, using the color mode to localize the deep palmar branch of right radial artery. The measurements were collected at the segment 5 mm distal to the radial styloid process. Due to slight wrist dorsiflexion, the desired segment deviated 20–30 degrees from the horizontal plane. The investigator steered the color window to 30 degrees and corrected the Doppler angle of insonation to less than 60 degrees for Doppler spectrum analysis. We averaged three waves at rest to represent the basal hemodynamic status and then asked the participants to produce a strong cough following a deep breath. An artifact caused by this muscle activity indicated a sufficient effort to excite the sympathetic system (Figure 1). After stimulation, there was a gradual decrease in peak blood flow velocity, and the wave of maximal change was taken as the outcome of sympathetic excitation. The SVR latency indicated the period between the artifact by inspiratory cough to the maximal deflection of Doppler wave forms (Figure 1). Unless the Doppler signal was missed due to hand movement, the test was not repeated to avoid fatigue or habituation. When a repeat was necessary, the participant was given at least 5 minutes of rest.

 Pulsatility index (PI) was used to indirectly depict vascular resistance, derived from the following calculation: *V*_max⁡_– *V*_min⁡_/*V*_max⁡ mean_ [13]. *V*_max⁡_ is the peak systolic velocity, and *V*_min⁡_ is the minimum forward diastolic velocity in unidirectional flow or the maximum negative velocity in diastolic reversal. *V*_max⁡ mean_ and *V*_mean_ are the maximal and mean flow velocity averaged over 1 cardiac cycle, respectively. The arterial diameters were obtained under duplex ultrasound mode with dual imaging, coincident with the peak systolic velocity on the Doppler spectrum. The vessel diameter was determined by Image J (National Institutes of Health, 9000 Rockville Pike, Bethesda, MD 20892), measuring from the outer vessel wall to the opposite inner wall. The blood flow volume per second was calculated as follows: (diameter/2)^2^ × *V*_mean_ × π. The heart rate in beats per minute was calculated to be 60 seconds divided by the peak-to-peak interval during 1 cardiac cycle.

### 2.3. Head-Up Tilt Protocol

The dopaminergic medication and therapy for OH were withheld 12 hours before the test. The study was carried out 15 minutes following the SVR test. Blood pressure and heart rate were recorded by a tonometric device (TANGO, SunTech Medical Instruments, Inc., NC, USA) placed at the brachial artery distal to right elbow crease. Mean arterial pressure was defined as two-third of diastolic blood pressure plus one-third of systolic blood pressure. The blood flow and arterial diameters of right common femoral artery were assessed at a 3-minute interval at the point 2 cm proximal to its bifurcation by the same ultrasound machine. The value of the concurrent mean arterial pressure divided by the blood flow represented vascular resistance of right femoral artery. Five-minute supine rest was required prior to the initial 5 minutes of baseline measurement. Then the electrically powered tilt table was tilted up to 60 degrees within 15 seconds for 10 minutes and returned to the horizontal plane with continuous observation for additional 5 minutes. If the participant complained neurological or cardiovascular discomfort related to fall of blood pressure, he would be reclined to the supine position immediately without completing 10 minutes of head-up tilt.

### 2.4. Statistic Methods

The hemodynamic variables and basic characteristics between controls and MSA patients were analyzed by using Mann-Whitney *U* test. We compared the parameters before and after cough or before and after head-up tilt by employing repeated measures analysis of variance. The association of measurements between SVR and the tilt table test was explored by Pearson's correlation methods. All the analyses were performed by using SPSS software version 12 (SPSS, IBM Corporation, Somers, NY). Statistical significance was assumed when *P* values were less than 0.05.

## 3. Results

**Figure 2:**
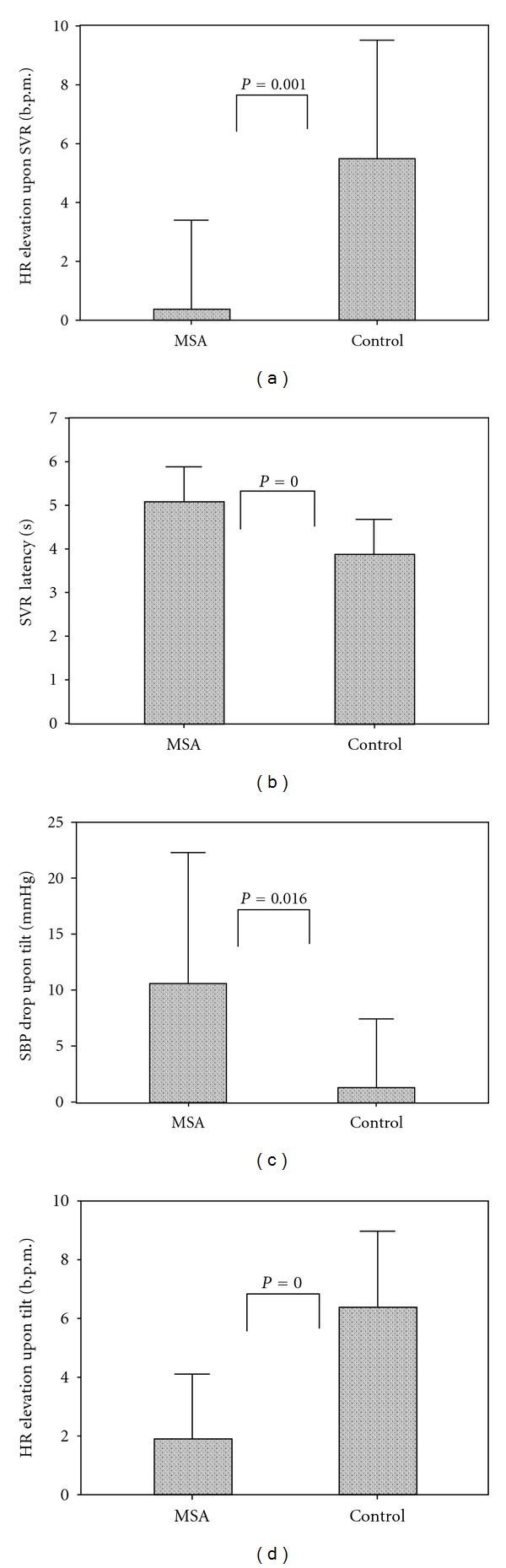
The comparisons of hemodynamic variables between MSA patients and healthy controls. Regarding SVR, MSA patients had (a) lower HR elevation and (b) a prolonged SVR latency. As for the tilt table study, MSA patients had (c) significant drop of SBP and (d) less HR elevation. Note: SVR: sympathetic vasomotor response; MSA: multiple system atrophy; HR: heart rate; SBP: systolic blood pressure.

The age, body weight, and height appeared alike in healthy controls and MSA patients (Table 1). Regarding the hemodynamic variables measured during SVR, the flow velocity, pulsatility index, vascular diameter, and heart rate did not differ between groups at rest and after sympathetic stimuli. The inspiratory cough contributed to the decrement in velocity-associated parameters (*V*_max⁡_, *V*_min⁡_, *V*_max⁡ mean_, and *V*_mean_) and blood flow and the elevation in pulsatility index in both groups. However, the corresponding heart rate changes after sympathetic stimulation were significantly higher in controls, whereas the SVR latency was more prolonged in MSA patients (Table 2, Figure 2).

During the tilt table test, five MSA patients fulfilled the criteria of OH [14]. The supine systolic blood pressure was lower in healthy controls, while that dropped significantly in MSA patients after tilting up. No significant difference in peripheral vascular resistance and its associated change was identified between both groups. Nevertheless, MSA patients had a significantly reduced heart rate elevation upon head-up tilt compared to the values in controls (Table 3, Figure 2).

**Figure 3:**
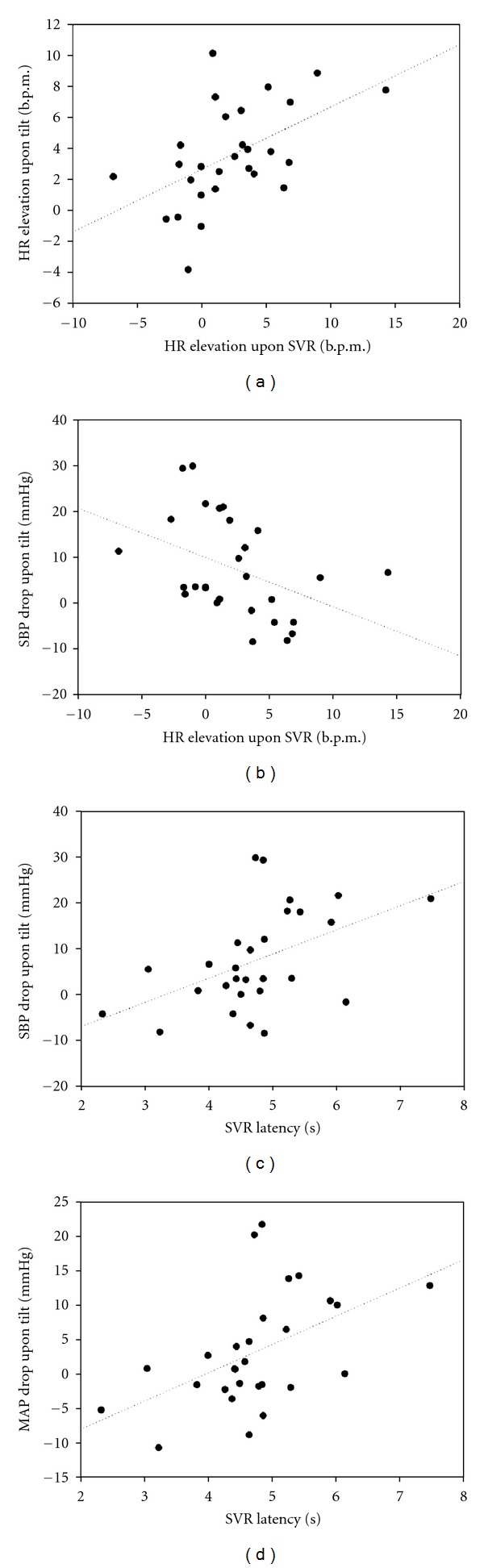
The correlation analysis of hemodynamic variables between SVR and the tilt table study. (a) The HR elevation upon SVR correlated positively with the HR elevation upon head-up tilt (*r* = 0.520, *P* = 0.005) but (b) negatively with the SBP drop from supine to tilt (*r* = −0.414, *P* = 0.029). The SVR latency demonstrated a positive correlation with (c) the SBP drop and (d) MAP drop after tilting up (*r* = 0.486, *P* = 0.009 and *r* = 0.5, *P* = 0.007, resp.). Note: SVR: sympathetic vasomotor response; HR: heart rate; SBP: systolic blood pressure; MAP: mean arterial pressure.

Regarding correlation analysis, we found that the change of heart rate in SVR correlated positively with the change of heart rate during head-up tilt (*r* = 0.520, *P* = 0.005) but negatively with the difference of systolic blood pressure from supine to tilt (*r* = −0.414, *P* = 0.029). The latency in SVR exhibited similar tendency, demonstrating a positive correlation with the drop of systolic and mean blood pressure during head-up tilt (*r* = 0.486, *P* = 0.009 and *r* = 0.5, *P* = 0.007, resp.) (Figure 3).

## 4. Discussion

This study employed PWD to investigate the hemodynamic alternations upon SVR between healthy participants and patients with possible MSA. Patients with MSA presented with a prolonged SVR latency and a reduced magnitude of heart rate acceleration following sympathetic stimulation. Compared with the measurements in the tilt table test, the elevation of heart rate upon SVR was positively proportional to the increase of heart rate after head-up tilt. The correlation analysis indicated that the degrees of blood pressure drop during postural challenge was positively associated with the SVR latency, but negatively correlated with its corresponding heart rate change upon SVR.

 In this study, inspiratory cough during SVR accelerated heart rates significantly more in healthy controls than in MSA patients, whereas no statistical difference was identified between both groups regarding parameters like pulsatility index, velocity, and flow. Previous studies using continuous wave Doppler ultrasound to assess SVR in patients with diabetic neuropathy or carpal tunnel syndrome indicated that reduced elevations in pulsatility or resistive index after stimulation implied impairment in the corresponding sympathetic circuits [15, 16]. However, for patients with MSA, the deficit in sympathetic neurotransmission was known to be preganglionic, therefore the indicators for peripheral vasoconstriction like pulsatility index may remain normal as controls [3]. Another study recruited only patients with severe OH, revealing more skin flow reduction measured by LDF in MSA patients than in healthy participants, which was not consistent with our findings [17]. In our study, only 5 patients met the criteria of OH, implying that most patients were in their early period of MSA. We speculate that peripheral vasoconstriction may be eventually hampered as the disease progresses, but is preserved in the initial stage. On the other hand, since SVR is commonly used to evaluate integrity of peripheral sympathetic pathways [5, 10], none of antecedent research measures the concurrent heart rate changes whose abnormality may appear earlier than other parameters. Because the SVR latency measured by PWD correlates with the R-R interval of heart beats, a prolonged latency and reduced heart rate elevation in MSA patients both suggest that their initial deficit in sympathetic circuits exists in the central level. 

 Our result showed that MSA patients exhibited a reduced magnitude of heart rate elevation upon head-up tilting, which was positively correlated with the heart rate change observed upon SVR. Elevation of heart rate mediated by baroreflex is a normal physiological response against orthostatic stress. The reflex loop is responsible for the prompt withdrawal of parasympathetic inhibition and subsequent augmentation of sympathetic outflows once the baroreceptors sense the blood pressure decrease. Previous research reported that baroreflex sensitivity was blunted in MSA patients and the impairment was related to the disease severity [18]. Another study indicated that MSA patients with OH had lower baroreflex cardiovagal gain than healthy controls [19]. Regarding the sympathetic provocative maneuver used in SVR, the inspiratory gap before the forceful cough reduced intrathoracic pressure, promoted venus return, and sensitized the baroreceptor by temporary blood pressure drop. Thus we suppose that heart rate change upon SVR shares a similar neurophysiological mechanism as that in the tilt table study, and the inadequate responsiveness in both tests mainly results from the deficiency of baroreflex.

 In this study, the systolic blood pressure was higher in MSA patients in supine position but dropped more after tilting up. We also found that the drop of blood pressure correlated positively with the period of SVR latency but negatively with the magnitude of heart rate elevation during SVR and head-up tilt. Supine hypertension appears common in MSA patients and is usually derived from medication against OH [20], whereas significantly greater blood pressure decline than that in controls implied the dysfunction in autonomic systems. Two major mechanisms account for maintaining blood pressure during orthostatic stress, including boosting vascular resistance or raising heart rates to compensate stroke volume depletion. Since the changes of femoral artery resistance did not differ between the patients and controls (Table 3), we assumed that OH in patients with MSA resulted from failure of heart rate elevation. The results of SVR and the tilt table study implied that the latency and heart rate change upon SVR were useful to predict blood pressure responses upon head-up tilt.

 The results may have some clinical implications. First, though previous research using 45 degrees head-up tilt found similar heart rate changes between MSA patients and controls [4], our study showed significant difference in heart rate elevation after tilting up to 60 degrees. This indicates that 60 degrees of tilt is more sensitive to detect the heart rate responsiveness upon orthostatic stress. For patients who are concerned about the posture-induced discomfort, using PWD to assess SVR can be considered as an alternative. Second, simultaneously monitoring of heart rates calculated by the wave to wave interval in the Doppler spectrum allows the assessment of central components of SVR upon sympathetic provocative tests. Our data also suggested that MSA patients had more severe impairment in central sympathetic activation than peripheral vasoconstriction.

There were some limitations in this study. Regarding the participants, males are included and difference in autonomic systems is known to exist between genders [21]. Therefore, the results of this study may not be generalized to female patients with MSA. Additionally, we chose postinspiratory cough as the sympathetic provocative maneuver, which might not be well performed in patients with movement disorder. For this reason, we only selected data with a visible transient flow velocity rise, which indicated sufficient efforts to excite sympathetic nerves. Moreover, it should be better to use multivariate analysis to explore the relationships between each hemodynamic variable. Nevertheless, we merely employed simple correlation methods since the statistical significance might be diluted when applying multivariate analysis on such small numbers of participants.

 In conclusion, our data suggested that patients with MSA had a prolonged SVR latency and reduced heart rate elevation after inspiratory cough. The blunted heart rate response upon SVR correlated with the magnitude of blood pressure drop in the tilt table test, leading SVR measured by PWD to become a useful alternative to predict the response upon orthostatic stress in MSA patients. Further study focusing on patients with Parkinsonism or other extrapyramidal syndromes is needed to evaluate the usefulness of SVR.

## Figures and Tables

**Table 1:** Basic characteristics in patients with multiple system atrophy (MSA) and healthy controls.

	MSA (*n* = 18)	Control (*n* = 10)	*P* values (Mann-Whitney *U* test)
Age (year)	65.1 ± 9.3	60.3 ± 7.0	0.165
Body height (cm)	166.1 ± 6.7	166.1 ± 7.2	0.660
Body weight (kg)	71.9 ± 10.4	68.4 ± 8.0	0.452
Disease duration (year)	3.3 ± 1.8		
Hoehn-Yahr stage	3.1 ± 0.9		
UMSARS I	14.9 ± 6.8		
UMSARS II	28.4 ± 12.1		
UMSARS IV	1.2 ± 1.2		

Note: Values are mean ± S.D.; UMSARS: Unified Multiple System Atrophy Rating Scale.

**Table 2:** The hemodynamic variables of sympathetic vasomotor response and their corresponding changes at baseline and after inspiratory cough.

	MSA (*n* = 18)	Control (*n* = 10)	*P* value: MSA versus control (Mann-Whitney *U* Test)
*Baseline*			
Pulsatility index	5.3 ± 3.4^‡^	4.4 ± 2.5^‡^	0.464
*V*_max⁡_ (cm/s)	39.0 ± 11.4^‡^	46.2 ± 19.0^‡^	0.356
*V*_min⁡_ (cm/s)	−0.5 ± 4.5^‡^	0.5 ± 6.4^‡^	0.436
*V*_max⁡ mean_ (cm/s)	10.1 ± 5.6^‡^	14.1 ± 10.2^‡^	0.408
*V*_mean_ (cm/s)	5.2 ± 3.1^‡^	7.0 ± 5.2^‡^	0.436
Flow amount (mL/min)	20.8 ± 17.6^‡^	30.8 ± 31.2^‡^	0.494
Diameter (mm)	2.7 ± 0.4	2.7 ± 0.5	0.944
Heart rate (beat/min)	79.6 ± 14.2	73.0 ± 9.7^‡^	0.249
*After inspiratory cough*			
Pulsatility index	7.8 ± 2.2^‡^	8.1 ± 2.0^‡^	0.654
*V*_max⁡_ (cm/s)	33.3 ± 9.8^‡^	35.6 ± 12.9^‡^	0.654
*V*_min⁡_ (cm/s)	−7.1 ± 2.9^‡^	−7.2 ± 3.5^‡^	0.906
*V*_max⁡ mean_ (cm/s)	4.8 ± 3.7^‡^	5.5 ± 2.3^‡^	0.944
*V*_mean_ (cm/s)	2.6 ± 0.9^‡^	2.7 ± 1.0^‡^	0.944
Flow amount (mL/min)	8.4 ± 5.2^‡^	8.4 ± 4.8^‡^	0.944
Diameter (mm)	2.4 ± 0.4	2.4 ± 0.3	0.981
Heart rate (beat/min)	80.1 ± 14.4	78.6 ± 8.5^‡^	0.981
*Difference between baseline and after inspiratory cough*			
Pulsatility index	2.5 ± 1.7	3.6 ± 1.8	0.133
Δ*V*_max⁡_ (cm/s)	5.7 ± 5.7	10.6 ± 10.9	0.454
Δ*V*_min⁡_ (cm/s)	6.5 ± 5.3	7.7 ± 5.2	0.654
Δ*V*_max⁡ mean_ (cm/s)	5.2 ± 4.2	8.5 ± 8.8	0.621
Δ*V*_mean_ (cm/s)	2.5 ± 2.3	4.3 ± 4.5	0.524
ΔFlow amount (mL/min)	12.0 ± 13.0	22.4 ± 26.9	0.408
ΔDiameter (mm)	0.2 ± 0.4	0.2 ± 0.4	0.832
ΔHeart rate (beat/min)	0.4 ± 3.0*	5.5 ± 4.0*	0.001*
Latency (s)	5.1 ± 0.8*	3.9 ± 0.8*	0.000*

Note: Values are mean ± S.D.; MSA: multiple system atrophy; ^‡^ or * indicated significant difference of hemodynamic measurements between baseline and after inspiratory cough or between MSA and control, respectively.

**Table 3:** The hemodynamic variables of the tilt table study and their corresponding changes at supine and after head-up tilt.

	MSA (*n* = 18)	Control (*n* = 10)	*P* value: MSA versus control (Mann-Whitney *U* Test)
*Supine*			
Systolic blood pressure (mmHg)	106.6 ± 6.9*	119.2 ± 16.4^‡∗^	0.016*
Diastolic blood pressure (mmHg)	69.0 ± 6.4	71.9 ± 9.6	0.408
Mean blood pressure (mmHg)	81.5 ± 6.1	87.7 ± 11.4^‡^	0.191
Heart rate (beat/min)	66.7 ± 8.0^‡^	74.9 ± 11.2^‡^	0.064
Peripheral vascular resistance (mmHg·mL^−1^·s^−1^)	27.1 ± 8.2^‡^	24.2 ± 6.0^‡^	0.588
*After head-up tilt*			
Systolic blood pressure (mmHg)	105.2 ± 11.6	108.5 ± 17.4^‡^	0.588
Diastolic blood pressure (mmHg)	71.5 ± 9.3	68.9 ± 11.5	1.000
Mean blood pressure (mmHg)	82.7 ± 9.8	82.1 ± 13.1^‡^	0.759
Heart rate (beat/min)	73.2 ± 7.2^‡^	76.9 ± 11.8^‡^	0.356
Peripheral vascular resistance (mmHg·mL^−1^·s^−1^)	52.5 ± 17.1^‡^	46.6 ± 11.6^‡^	0.759
*Difference between baseline and after head-up tilt*			
ΔSystolic blood pressure (mmHg)	1.4 ± 6.0*	10.7 ± 11.5*	0.045*
ΔDiastolic blood pressure (mmHg)	−2.5 ± 4.5	3.0 ± 7.7	0.064
ΔMean blood pressure (mmHg)	−1.1 ± 4.9	5.5 ± 8.7	0.057
ΔHeart rate (beat/min)	6.4 ± 2.6*	1.9 ± 2.2*	0.000*
ΔPeripheral vascular resistance (mmHg·mL^−1^·s^−1^)	25.4 ± 14.3	22.3 ± 10.6	0.944

Note: Values are mean ± S.D.; MSA: multiple system atrophy; ^‡^ or * indicated significant difference of hemodynamic measurements between baseline and after head-up tilt or between MSA and control, respectively.
